# Current status of the analytical validation of next generation sequencing applications for pharmacogenetic profiling

**DOI:** 10.1007/s11033-023-08748-z

**Published:** 2023-10-03

**Authors:** Tatjana Huebner, Michael Steffens, Catharina Scholl

**Affiliations:** Research Division, Federal Institute for Drugs and Medical Devices (BfArM), Kurt-Georg-Kiesinger-Allee 3, Bonn, 53175 Germany

**Keywords:** Analytical validation, Next generation sequencing, NGS, Whole exome sequencing, WES, Whole genome sequencing, WGS, Third generation sequencing, Pharmacogenomics, PGx, Profiling, Performance

## Abstract

**Background:**

Analytical validity is a prerequisite to use a next generation sequencing (NGS)-based application as an in vitro diagnostic test or a companion diagnostic in clinical practice. Currently, in the United States and the European Union, the intended use of such NGS-based tests does not refer to guided drug therapy on the basis of pharmacogenetic profiling of drug metabolizing enzymes, although the value of pharmacogenetic testing has been reported. However, in research, a large variety of NGS-based tests are used and have been confirmed to be at least comparable to array-based testing.

**Methods and Results:**

A systematic evaluation was performed screening and assessing published literature on analytical validation of NGS applications for pharmacogenetic profiling of *CYP2C9*, *CYP2C19*, *CYP2D6*, *VKORC1* and/or *UGT1A1*. Although NGS applications are also increasingly used for implementation assessments in clinical practice, we show in the present systematic literature evaluation that published information on the current status of analytical validation of NGS applications targeting drug metabolizing enzymes is scarce. Furthermore, a comprehensive performance evaluation of whole exome and whole genome sequencing with the intended use for pharmacogenetic profiling has not been published so far.

**Conclusions:**

A standard in reporting on analytical validation of NGS-based tests is not in place yet. Therefore, many relevant performance criteria are not addressed in published literature. For an appropriate analytical validation of an NGS-based qualitative test for pharmacogenetic profiling at least accuracy, precision, limit of detection and specificity should be addressed to facilitate the implementation of such tests in clinical use.

**Supplementary Information:**

The online version contains supplementary material available at 10.1007/s11033-023-08748-z.

## Introduction

In recent years, regulatory adaptations in the United States of America (USA) and the European Union (EU) with regard to the use of in vitro diagnostic applications in the management of drug therapy have been introduced [1, 2]. This also affected the performance requirements of next generation sequencing (NGS).

Analytical validity is considered a critical step in test assessment. According to the US in vitro diagnostic (IVD) regulation and the new in vitro diagnostic medical devices regulation (IVDR) in the EU, the analytical and clinical performance of an in vitro diagnostic test to be legally marketed should first be evaluated [3, 4]. Therefore, the suitability of an in vitro diagnostic NGS test needs to be demonstrated for the intended use [5, 3]. Laboratories offering targeted NGS [6], whole exome sequencing (WES) and whole genome sequencing (WGS) as diagnostic tests are also obliged to evaluate performance characteristics and carry out a full validation prior to clinical application as laboratory developed test (LDT). In the EU, the evaluation of analytical performance of an IVD or LDT should encompass a variety of general criteria also including the collection and handling of according specimen. However, explicit criteria that meet the needs of the assessment of analytical NGS performance are not specified [3]. Furthermore, standardization for performance evaluation is currently controversial [2].

For the analytical validation of targeted NGS based on NGS panels, in a recent publication on the consensus recommendation of the Association for Molecular Pathology and College of American Pathologists, an assessment of accuracy in terms of the positive percent agreement (PPA) and positive predictive value (PPV) was recommended. Furthermore, the precision of variant detection in terms of reproducibility and repeatability, reportable range and reference range, limits of detection, analytical specificity (interfering substances) and carryover was included in the recommendation [7].

In the USA, further guidance for analytical validation of in vitro diagnostics on the basis of NGS is provided by non-binding recommendations of the Food and Drug Administration (FDA). Here, test performance characteristics such as accuracy also in terms of PPA, technical PPV and additionally negative percent agreement (NPA), precision, limit of detection, and analytical specificity in terms of interference, cross-reactivity and cross-contamination are suggested for evaluation [8].

FDA documents such as the “Summary of Safety and Effectiveness” provide publicly available analytical performance evaluations of NGS-based diagnostic tests as companion diagnostics. However, predominantly for personalized therapeutic management for drugs applied in oncology [9]. In the USA, such approved NGS-based tests can therefore be applied in clinical practice for the intended use. However, no NGS-based companion diagnostic is approved to guide therapy with regard to the pharmacogenetic profile of drug metabolizing enzymes. Large-scale analyses such as whole exome and whole genome sequencing are increasingly used as laboratory developed tests [5]. Such data is considered a valuable resource for pharmacogenomic profiling [10–12]. However, the added value of WES and WGS applications compared to other large scale molecular genetic tests in pharmacogenetics is controversial and limitations were reported with regard to coverage and short read-based assessment [12, 13]. The status of the validation of NGS-based applications for clinical pharmacogenomic (PGx) profiling is unclear, although value for therapeutic management has been reported [10, 14], and clinical implementation studies have been performed in several countries [15–17]. Also in the EU, for a few drug prescriptions, e.g. testing for *cytochrome P450 2C1*9 (*CYP2C19*) variants prior to therapy with atazanavir is recommended and testing for *cytochrome P450 2D6* (*CYP2D6*) prior to therapy with eliglustat is even required [18]. However, PGx testing is not applied as companion diagnostics yet [9]. Approaches to repurpose e.g. whole exome sequencing data obtained in clinical settings also for pharmacogenetic profiling suggest that such secondary findings may provide an extraordinary opportunity to integrate valuable information for personalized treatment. However, the feasibility evaluations reported limitations with regard to several pharmacogenes [19, 20]. Furthermore validity of these approaches needs to be established [20].

Here, on the basis of current literature identified at the platforms Pubmed and Pubmed Central, we provide an overview of the available information on the analytical validation status of applied targeted NGS including second and third generation sequencing and furthermore whole exome and whole genome sequencing for targeting relevant pharmacogenetic biomarkers. Additionally, we discuss the potential of third generation sequencing techniques for whole genome sequencing and the assessment of pharmacogenetic information provided by such techniques.

## Methods

The FDA list “Nucleic acid-based tests” was applied in order to analyze the validation status of NGS applications for genetic testing of pharmacogenes coding drug metabolizing enzymes. Here, listed genes of the category “drug metabolizing enzymes” were used for further investigations [21]. The list provides genetic tests that have either been cleared or approved. Such tests are appropriate for comparison in the evaluation of the analytical validation of other testing techniques for pharmacogenetic profiling. Second generation and third generation sequencing techniques were included in the analysis.

### Analytical validation on the basis of cleared or approved genetic tests

Screening for publications providing a performance comparison of FDA approved nucleic acid-based diagnostic tests concerning drug metabolizing enzymes with second and third generation sequencing applications was performed in August 2022. Many of these tests were also CE certified in the European Union. The aim was to identify whether such approved or cleared tests have been used to validate NGS based applications with a focus on relevant pharmacogenes. According to the FDA recommendations, such tests identified as appropriate by the FDA, should be applied as reference tests of choice in an analytical validation [8]. Here, the publication platforms Pubmed and Pubmed Central were screened for according publications using the keywords “test name” (Table 1, see Trade Name), associated gene(s) (Table 1, see “Gene”), next generation sequencing or whole exome sequencing or whole genome sequencing or long read sequencing or Nanopore or Pacific Biosciences and validity or validation. Publications identified for second and third generation sequencing applications were evaluated separately. Congress abstracts were not included and duplications were excluded. Publications not providing information on validation results with the according FDA cleared or approved nucleic acid-based diagnostic tests involving the according gene in the method or result section of the publication were excluded. The few publications included were analyzed for the evaluation of NGS performance on the basis of relevant analytical validation criteria recommended for NGS in current literature such as accuracy, precision, analytical specificity (endogenous and exogenous interference, cross-reactions, cross-contamination) and limit of detection [7, 8].

**Table 1:** Test search with the according keywords (trade name, gene(s), NGS application, validity or validation) on the basis of the FDA list “Nucleic acid-based tests” focusing on the category “drug metabolizing enzymes”

Gene	Trade Name/	Manufacturer
	method	
**CYP2D6**	xTAG CYP2D6 Kit v3/	Luminex Molecular Diagnostics, Inc.
	Multiplex PCR followed by multiplex ASPE for genotyping, hybridized to multiplexed fluoresceing microparticles, detected by flow cytometry	
**CYP2C19**	Spartan RX CYP2C19 Test System/ Microarray	Akonni Biosystems Inc.
**CYP2C9, VKORC1**	TruDiagnosis System, TruArray® Warfarin Sensitivity Test Kit/	Akonni Biosystems Inc
	Microarray	
**CYP2C19**	Verigene CYP2C 19 Nucleic Acid Test/	Nanosphere, Inc
	Microarray	
**CYP2C19**	INFINITI CYP2C19 Assay/	AutoGenomics, Inc.
	Microarray	
**UGT1A1**	Invader UGT1A1 Molecular Assay/	Third Wave Technologies Inc.
	Microarray	
**CYP2C19**	Roche AmpliChip CYP450 microarray/	Roche Molecular Systems, Inc.
	Microarray	
**CYP2C9, VKORC1**	eSensor Warfarin Sensitivity Saliva Test/	GenMark Diagnostics
	Microarray	
**CYP2C9, VKORC1**	eQ-PCR LC Warfarin Genotyping kit/	TrimGen Corporation
	Realtime PCR	
**CYP2C9, VKORC1**	eSensor Warfarin Sensitivity Test and XT-8 Instrument/	Osmetech Molecular Diagnostics
	Microarray	
**CYP2C9, VKORC1**	Gentris Rapid Genotyping Assay – CYP2C9 & VKORCI/	ParagonDx, LLC
	Realtime PCR	
**CYP2C9, VKORC1**	INFINITI 2C9 & VKORC1 Assay for Warfarin/	AutoGenomics, Inc.
	Microarray	
**CYP2C9, VKORC1**	Verigene Warfarin Metabolism Nucleic Acid Test and Verigene System/Microarray	Nanosphere, Inc.
	(Gold nanoparticles hybridization technology)	

ASPE: Allele Specific Primer Extension

### Validation status of NGS based applications

To further evaluate the validation status of targeted NGS, WES and WGS in research and the clinic with regard to the previously analyzed pharmacogenes (Table 1), a second keyword search was performed. Cytochrome P450 2C9, 2C19 and 2D6, Vitamin K epOxide Reductase Complex Subunit 1 (VKORC1) and UDP-Glucuronosyltransferase 1 Polypeptid A1 (UGT1A1) were also the focus of this evaluation. This search was extended to any test or orthogonal method applied for performance comparison with an NGS based application to increase the yield of findings. Thereby, the keywords next generation sequencing and *VKORC1* or *CYP2C9* or *CYP2C19* or *CYP2D6* or *UGT1A1* and validity or validation were used to screen the publication platforms Pubmed and Pubmed Central. The same search as for NGS was performed for WES and WGS. Title and abstract were screened for suitability of the publication in terms of information on a performance evaluation of the according method involving the according gene of interest. The included publications were analyzed for the evaluation of analytical validation criteria recommended for NGS in current literature such as limit of detection, accuracy (positive percent agreement, negative percent agreement and positive predictive value), precision (reproducibility and repeatability), and analytical specificity (endogenous and exogenous interference, cross-reactions, cross-contamination).

## Results

### Analytical validation of NGS- based tests applying orthogonal FDA cleared or approved genetic tests

**Fig. 1:**
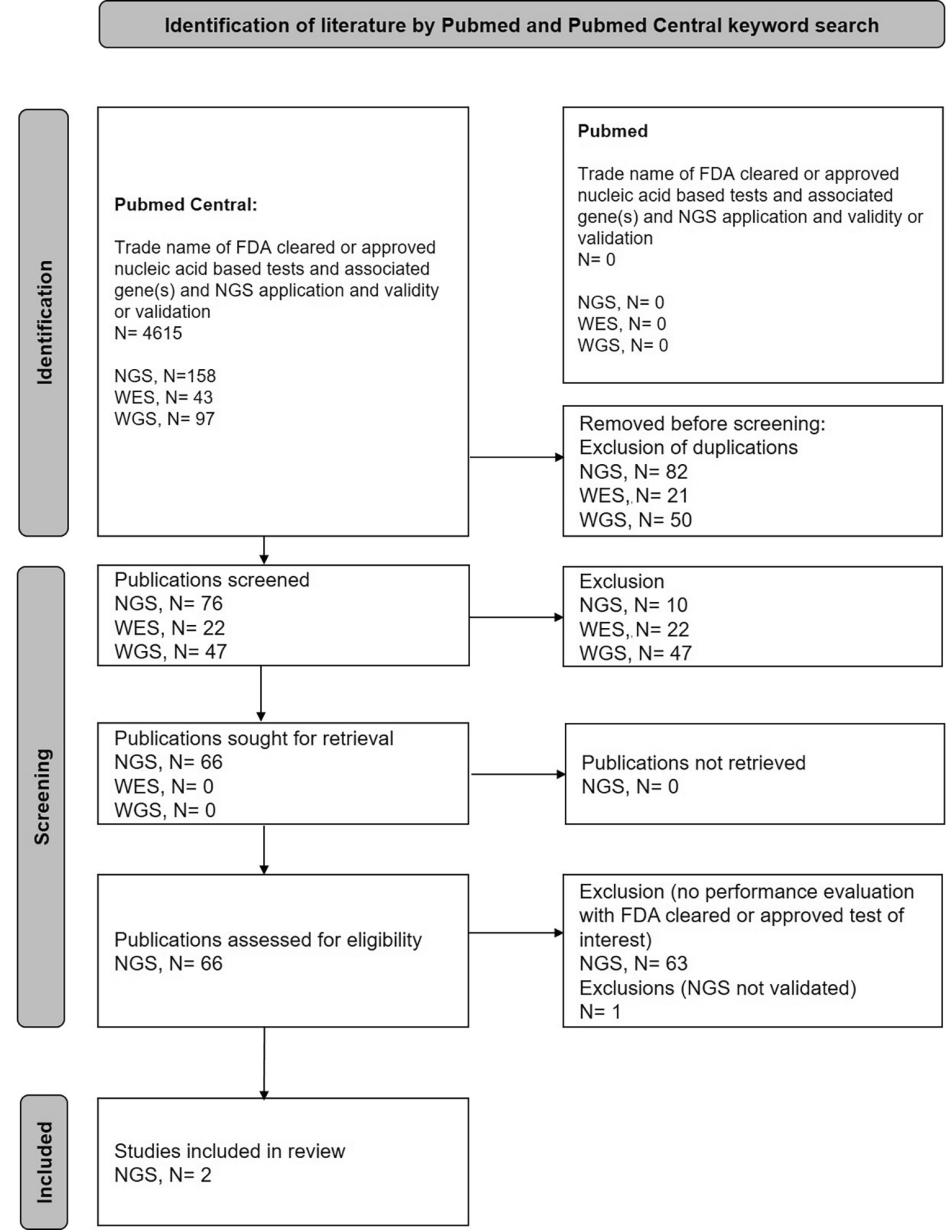
PRISMA 2020 scheme of the literature search for analytical performance studies of NGS-applications with FDA cleared or approved orthogonal tests in Pubmed and Pubmed Central

The present keyword search (Fig. 1) aimed to identify published analytical validation studies of NGS-applications with FDA cleared or approved orthogonal tests.

FDA cleared or approved genetic tests used for keyword search were identified as PCR- or predominantly microarray-based methods for genotyping of drug metabolizing enzymes (Table 2). A keyword search applying the platform Pubmed did not result in any findings. An additional keyword search on the platform Pubmed Central resulted in a few publications addressing some of the cleared or approved nucleic acid-based tests. The keywords validation and validity resulted in overlapping publication lists. However, few suitable publications with regard to test comparison or validation were identified (Table 2) [22, 23].

**Table 2:** Test search results with the according keywords (trade name, gene(s), NGS application, validity or validation). All findings for second generation sequencing (SGS) including targeted NGS, WES and WGS and for third generation sequencing (TGS) were summed up excluding duplications. The number of according publications providing performance evaluations was included in brackets.

Gene	Trade Name/method	Manufacturer	Second generation sequencing	Third generation sequencing	Validation performed
**CYP2D6**	xTAG CYP2D6 Kit v3/Multiplex PCR followed by multiplex ASPE for genotyping, hybridized to multiplexed fluoresceing microparticles, detected by flow cytometry	Luminex Molecular Diagnostics, Inc.	4 (**1**)	3 (**1**)	SGS: Carvalho Henriques et al (2021) Only concordance evaluation TGS: Qiao et al. (2016) Performance evaluation including quality metrics, concordance and precision
**CYP2C19**	Spartan RX CYP2C19 Test System/Microarray	Akonni Biosystems Inc.	7	-	No
**CYP2C9, VKORC1**	TruDiagnosis System, TruArray® Warfarin Sensitivity Test Kit/Microarray	Akonni Biosystems Inc	-	-	No
**CYP2C19**	Verigene CYP2C 19 Nucleic Acid Test/Microarray	Nanosphere, Inc	-	-	No
**CYP2C19**	INFINITI CYP2C19 Assay/Microarray	AutoGenomics, Inc.	17	6	No
**UGT1A1**	Invader UGT1A1 Molecular Assay/Microarray	Third Wave Technologies Inc.	19 (**1**)	5	SDS: Tang et al. (2021) No focus on NGS applications
**CYP2C19**	Roche AmpliChip CYP450 microarray/Microarray	Roche Molecular Systems, Inc.	19	7	No
**CYP2C9, VKORC1**	eSensor Warfarin Sensitivity Saliva Test/Microarray	GenMark Diagnostics	-	-	No
**CYP2C9, VKORC1**	eQ-PCR LC Warfarin Genotyping kit/Realtime PCR	TrimGen Corporation	-	-	No
**CYP2C9, VKORC1**	eSensor Warfarin Sensitivity Test and XT-8 Instrument/Microarray	Osmetech Molecular Diagnostics	-	-	No
**CYP2C9, VKORC1**	Gentris Rapid Genotyping Assay – CYP2C9 & VKORCI/Realtime PCR	ParagonDx, LLC	2	2	No
**CYP2C9, VKORC1**	INFINITI 2C9 & VKORC1 Assay for Warfarin/Microarray	AutoGenomics, Inc.	10	3	No
**CYP2C9, VKORC1**	Verigene Warfarin Metabolism Nucleic Acid Test and Verigene System/Microarray (Gold nanoparticles hybridization technology)	Nanosphere, Inc.	8	-	No

ASPE: Allele Specific Primer Extension

None of the publications in this literature search provided a comparison of WES or WGS with the listed nucleic acid-based tests. Publications of comparison experiments with next generation sequencing were identified in the keyword search for the Invader UGT1A1 Molecular Assay (Third Wave Technologies Inc., Wisconsin, USA) [24] and the xTAG CYP2D6 Kit v3 (Luminex Molecular Diagnostics, Inc., Toronto, ON, Canada) [22, 23]. The published performance comparison with the Invader UGT1A1 Molecular Assay however, was carried out with the OpenArray pharmacogenomics panel, which comprised of 4 customized TaqMan® OpenArray Genotyping Plates. NGS was included in the validation of the OpenArray panel (Thermo Fisher Scientific, Waltham, MA, USA) but was not validated with the Invader UGT1A1 Molecular Assay [24]. Therefore, this publication also did not result in an appropriate finding according to the inclusion criteria for this review. Performance comparison of the Luminex xTAG CYP2D6, with second generation sequencing was detected in only one of the identified publications [22]. Here, Carvalho Henriques et al. provided only an evaluation of concordance for *CYP2D6* and *CYP2C19* variant detection in their cross-validation of a large variety of different techniques. These also included NGS represented by Ion Torrent™semiconductor sequencing on the basis of the Ion AmpliSeq Pharmacogenomics Panel (Thermo Fisher Scientific, Waltham, MA, USA). Therefore, the accuracy via positive percent agreement, negative percent agreement and positive predictive value and also evaluations in terms of further important validation criteria such as precision, limit of detection, and analytical specificity were not addressed.

Performance comparison of the Luminex xTAG CYP2D6 with a third-generation sequencing application was only identified in one publication [23]. Qiao et al. provide a concordance evaluation of the long-read SMRT sequencing of *CYP2D6* on the Pacific Biosciences platform with the xTAG CYP2D6 Kit v3 (Luminex Corporation, TX, USA) and in terms of CNVs, the TaqMan® real-time qPCR Copy Number Assays (Applied Biosystems, Carlsbad, CA, USA). Furthermore, quality metrics and precision of the SMRT sequencing was evaluated in terms of intra- and inter-run reproducibility on the basis of triplicates. However, sample size was low and other aspects of accuracy such as positive percent agreement, negative percent agreement and positive predictive value and other criteria of analytical validation were not addressed [23].

### Validation status of NGS based applications

**Fig. 2:**
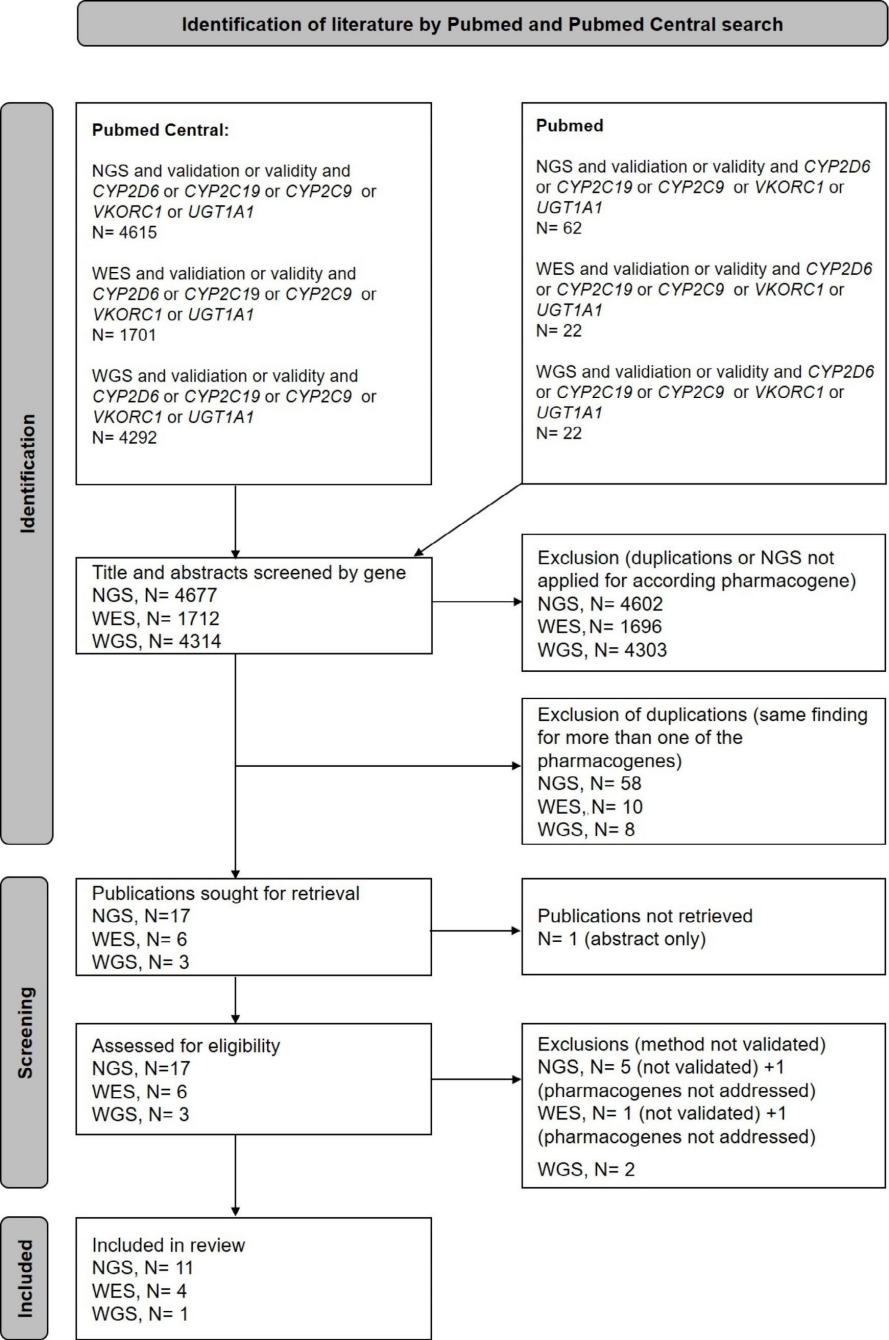
Scheme of the literature search for analytical performance evaluations of NGS-applications in Pubmed and Pubmed Central

In the second literature search described previously, about 99% of the publications screened by title and abstract were excluded, as they did not include information on sequencing of the pharmacogenes of interest or the use of NGS applications or any aspects of performance evaluation. Duplications due to overlapping results by applying the keywords validity or validation and due to published overlapping analyses including several of the pharmacogenes of interest were excluded in the screening phase. Further publications were excluded in the assessment for eligibility due to lacking focus on the NGS applications or the pharmacogenes of interest in the performance evaluations (Fig. 2).

In summary, of the 15 publications included (1 duplicate assessed for WGS and WES), 13 publications on second generation sequencing including also WES and WGS and two publications with a focus on third generation sequencing of *CYP2D6* were identified with performance evaluations. They provided information to target at least one of the pharmacogenes of interest (Table 3). All of these publications provide concordance evaluations of the next generation sequencing application with other genotyping methods such as TaqMan-based genotyping assays, Sanger sequencing, Agena Bioscience ADME genotyping panels, array-based applications and custom PCR-based assays (Supplementary material 1). The third generation sequencing articles identified used the Pacific Biosciences platform [23, 25]. Evaluations focusing on second generation sequencing were mainly based on the Illumina platforms, for WES and WGS the HiSeq 2000 and/or HiSeq 2500 platforms were used [26–28]. For targeted second generation sequencing, the HiSeq platforms (HiSeq 1500, HiSeq 2000, HiSeq 2500, HiSeq 4000) [29–32, 16] the MiSeq, NextSeq 500 and MiniSeq system was reported [33–35].

**Table 3:** Publications identified in the second literature search reporting approaches of analytical validation or concordance evaluation of NGS-based tests

Publication	Genotyped pharmacogenes of interest	Concordance evaluation	Further performance evaluation
**Second generation sequencing**			
Beaubier N et al. (2019)	*CYP2D6, UGT1A1*	yes	yes
Carvalho Henriques B et al. (2021)	*CYP2C19, CYP2D6*	yes	no
Chua EW et al. (2016)	*CYP2C19, CYP2D6*	yes	no
Ng D et al. (2017)	*VKORC1, CYP2C9, CYP2C19, CYP2D6, UGT1A1*	yes	no
Gulilat M et al. (2019)	*CYP2C9, CYP2C19, CYP2D6, UGT1A1*	yes	yes
Hamilton A et al. (2016)	*UGT1A1*	yes, no focus on pharmacogenes	no
Klein K et al. (2019)	*VKORC1, CYP2C9, CYP2C19, CYP2D6, UGT1A1*	yes	yes
Lee SB et al. (2022)	*VKORC1, CYP2C9, CYP2C19, CYP2D6, UGT1A1*	yes	no
Ramudo-Cela L et al. (2020)	*VKORC1, CYP2C9, CYP2C19, CYP2D6, UGT1A1*	yes	yes
Rasmussen-Torvik LJ et al. (2017)	*VKORC1, CYP2C9, CYP2C19, CYP2D6, UGT1A1*	yes	no
Silver A et al. (2022)	*VKORC1, CYP2C9, CYP2C19, CYP2D6, UGT1A1*	yes	yes
Xiang D et al. (2019)	*CYP2D6*	yes	NA
Yang W et al. (2016)	*VKORC1, CYP2C9, CYP2C19, CYP2D6, UGT1A1*	yes	yes
**Third generation sequencing**			
Buermans HP et al. (2017)	*CYP2D6*	yes	no
Qiao W et al. (2016)	*CYP2D6*	yes	yes

Library preparation systems used for test evaluations on the Illumina platforms also differed. The Agilent SureSelect kits were used more often than other enrichment kits (Supplementary material 1). Other platforms were used when only a few variants or genes were analyzed. The PyroMark Q24 (QIAGEN GmbH, Venlo, Netherlands)), a pyrosequencing platform, was applied in a comparison of CYP2D6*10 identification and the Ion Chef instrument along with the Ion AmpliSeq Pharmacogenomics Panel was used in a cross-validation of several molecular genetic techniques for genotyping of *CYP2D6* and *CYP2C19* (Supplementary material 1) [36, 22]. NGS applications assessed in a majority of the analysed publications (93%), considered the pharmacogene *CYP2D6* while *CYP2C9*, *CYP2C19*, *VKORC1* and *UGT1A1* were less often covered (Table 4). However, in one of the analyzed publications, the NGS application that covered *CYP2D6* and *UGT1A1* was an oncology sequencing assay and therefore not explicitly intended to be used for pharmacogenetic profiling (Supplementary material 1) [29].

**Table 4:** Overview of publications evaluating genetic tests based on second and third generation sequencing that include the according pharmacogenes of interest

Gene	Second-generation sequencing, (WES, WGS, NGS; N = 13 ex duplications)	Third-generation sequencing (N = 2)
CYP2C9	8	0
CYP2C19	10	0
CYP2D6	12	2 SMRT PacBio
UGT1A1	9	0
VKORC1	6	0

In addition to concordance evaluations, about 46.67% (7 of 15) of the analyzed publications provide further data on analytical performance criteria (Table 3). Accuracy assessments (33.3%, 5 of 15) were performed, these included overall accuracy [31] or in a majority of cases, as is recommended, accuracy in terms of the PPV along with positive percent agreement (also referred to as sensitivity) and negative percent agreement (referred to as specificity) (26.67%, 4 of 15) [29, 32, 35, 33] (Table 5). The limit of detection for variant types, such as SNVs and indels, is reported in one publication and was determined on the basis of detected variant allele fractions [29]. In 3 further publications only assessments in terms of minor allele frequency or fraction [31, 28] or detection rate [35] were performed without determining a limit of detection for the analyzed variant types (Supplementary material 2). Only one publication providing an evaluation of a targeted second generation sequencing panel appropriately addressed an evaluation of precision, in terms of intra-run and inter-run precision, that also provided the most comprehensive assessment in terms of FDA validation criteria such as also accuracy and further criteria such as reportable regions along with quality of the NGS metrics. However, analytical validation was based on only three reference samples [35]. Furthermore, one publication evaluating *CYP2D6* SMRT third generation sequencing reported an assessment of precision in terms of triplicate intra-run sequencing of a validation sample and inter-run sequencing [23].

**Table 5:** Publications identified in the second literature search, providing evaluations of performance criteria

Publication	Accuracy	Limit of detection	Analytical Specificity	Precision
**Second generation sequencing**				
Klein K et al. (2019)	overall accuracy	yes (minor allele frequency)	no	no
Beaubier N et al. (2019)	yes	via variant allele frequency	no	no
Ramudo-Cela L et al. (2020)	yes	no	no	sample triplicates only
Silver A et al. (2022)	yes (SNV, Indels)	detection rate	no	yes
Gulilat M et al. (2019)	yes	no	no	no
Yang W et al. (2016)	no	minor allele frequency	no	no
**Third generation sequencing**				
Qiao W et al. (2016)	no	no	no	yes

In summary, few studies evaluate analytical performance criteria such as limit of detection, accuracy and precision of second generation sequencing panels and fewer still focus on third generation sequencing. Analytical specificity in terms of interference, cross-reactions and cross-contamination has not been assessed or provided by any of these published evaluations. Therefore, according to the information provided on the platforms Pubmed and Pubmed Central, none of the NGS- based tests has an analytical performance evaluation that has been executed according to the current recommendations in literature and of the FDA. Although, several studies compare WES and WGS with other molecular genetic techniques in terms of variant detection in pharmacogenes coding drug metabolizing enzymes, here also no articles were identified that carried out an analytical validation in terms of evaluating performance criteria as recommended for NGS-based IVDs or LDTs [26, 30, 27, 28].

## Discussion

The present literature assessment shows that published studies on the analytical validation of NGS based pharmacogenetic tests concerning drug metabolizing enzymes are scarce. Specifically, this applies for WES, WGS and also third generation sequencing, as currently represented by Single Molecule Real Time sequencing using the PacBio (Pacific Biosciences of California, Inc, California, USA) and increasingly also Oxford Nanopore (Oxford Nanopore Technologies (ONT) Inc, Oxford, UK) platforms.

According to currently available, but non-binding, FDA recommendations, the evaluations identified are not sufficiently comprehensive. Furthermore, also, relevant analytical performance metrics according to the College of American Pathologists (CAP) guidance documents and the MM09 guideline of the Clinical and Laboratory Standards Institute (CLSI) were not addressed by the evaluated publications. In addition to the aforementioned analytical performance criteria recommended by the FDA, the CAP/ CLSI analytical performance metrics also include robustness, reportable range and reference interval. Metrics such as interference and cross-reactivity are listed in the CAP/CLSI MM09 test validation worksheet as IVD (not LDT) terminology only and cross-contamination is not addressed [37] (Supplementary material 3). Required analytical performance characteristics for IVDs in general, listed by the US Medicare, Medicaid, and Clinical Laboratory Improvement Amendments (CLIA) programs § 493.1253(c), comprise similar metrics to CAP/CLSI MM09 for IVDs, however not specified for NGS applications [38]. In the EU, required general analytical performance characteristics provided by the IVDR (Annex I chapter II 9.1 (a)) also include a larger scope and correspond to the CAP and CLIA criteria and cover also the specified FDA recommendations for NGS (Supplementary material 3). The performance metrics addressed by the evaluated publications, therefore do not meet CLIA and IVDR requirements as well. Such tests therefore are not suitable to be marketed and applied for clinical pharmacogenetic profiling in the USA or the EU. For an appropriate analytical validation of an NGS based qualitative test for pharmacogenetic profiling at least accuracy, precision, limit of detection and specificity should be assessed as recommended by the FDA guidance document “for Stakeholders and Food and Drug Administration Staff” [8] and as analyzed in this literature search. Furthermore, we suggest that also the reportable range and the range of outcomes expected in a normal population (normal interval) should be determined as recommended additionally by the CAP and the CLIA.

The most extensive performance evaluations were identified for targeted NGS panels that encompass a large variety of target genes, but without a direct focus on pharmacogenetic profiling. Here, Silver A et al. (2022) provided the best analytical validation in terms of the scope of performance criteria. The evaluation included accuracy and precision as recommended by the FDA and detection rates of targeted alleles, however did not include analytical specificity in terms of interference, cross-reactions and cross-contamination (Supplementary material 2). Furthermore, the reportable range was determined, which was however not a criterion of the present literature assessment. Ramudo-Cela L et al. (2020) evaluated accuracy of a targeted NGS panel according to FDA recommendations and partly precision in terms of reproducibility as sample triplicates were applied. The NGS panels however comprise a total of 430 (Silver A et al., 2022) and 389 (Ramudo-Cela L et al., 2020) genes and too large a panel size is not recommended as it can affect the efficiency of the laboratory, due to the complexity in interpreting the outcomes and the depth of coverage required [7]. Beaubier N et al. (2019) evaluated accuracy and LOD for different variant types as recommended by the FDA, however the intended use of the panel was to detect somatic alterations and microsatellite instability in solid tumors. It is therefore not validated for pharmacogenetic profiling although CYP2D6 and UGT1A1 were included in the panel. In the publication by Klein K et al. (2019), the lowest level of concordance with FDA recommendations was found with respect to the evaluation of performance criteria for a targeted NGS panel. Minor allele frequencies were addressed, however without determining detection limits and accuracy was evaluated as overall accuracy and not as recommended by the FDA via PPA, NPA and PPV. No further criteria were evaluated. Most of the publications assessed, include profiling of the highly polymorphic gene *CYP2D6* which is also a challenging locus to analyze due to complex structural variation.

A more comprehensive analysis of complex genetic information to assess the pharmacogenetic profile associated with the drug metabolism of a patient can be provided by WES and WGS [10]. In our literature search two publications were identified evaluating performance of WES or WES and WGS in terms of also the according pharmacogenes of interest (Supplementary material 2) [33, 28]. Both publications mainly focus on concordance analyses with orthogonal methods and quality metrics such as call rates or depth of coverage and not on the FDA analytical validation criteria. However, Gulilat M et al. (2019) evaluated accuracy of WES only without PPV as recommended and Yang W et al. (2016) addressed minor allele fractions only without reporting the determination of detection limits according to FDA guidance. Recommendations providing guidance on the analytical validation of WGS in clinical context were published in recent years. They indicate that traditional approaches with regard to the evaluation of performance metrics for the complete NGS based assay alone is not sufficient [39].

Large-scale massive parallel sequencing applications such as WES and WGS do not target specific genes, sequence contexts, diseases or associated variant types per se. Therefore, here the aim of analytical validation is to evaluate and determine reliable metrics for a suitable performance with regard to the largest percentage of area analyzed to assure high quality of sequencing results overall. It is intended that variant calling over the target region is sensitive and precise and also allows for the determination of regions or bases that fail requirements for appropriate variant calls [5].

Metrics considering aspects of genome complexity such as sequence content and the variety of different variant types should also be assessed, as variability in the accuracy of variant calling is also context-driven. Diagnostic accuracy is often evaluated in terms of sensitivity (PPA) and specificity (NPA). However, due to the expected large quantity of true negatives in WGS, precision is recommended as a more helpful metric for accuracy than specificity (NPA). In general, also for WGS, the assessment and extent of metrics as published in the non-binding guidance document for NGS by the FDA is recommended and the number of samples is suggested to be adapted to variant type or the analyzed region [39]. In the present literature search, the only publication evaluating performance of WGS for pharmacogenetic profiling did not address analytical performance criteria including precision as recommended. Current studies evaluating NGS-based test performance for pharmacogenetic profiling mainly provide concordance assessments with orthogonal methods (Supplementary material 2).

Compared to targeted NGS panels analytical sensitivity for exome sequencing is lower as depth of coverage is not uniform or insufficient affecting also analytical specificity and variant calling. Thus, Sanger Sequencing is needed to complete content and identify false positive calls [40].

For the profiling of pharmacogenes, so far, short read WGS has been proposed to be a more suitable NGS application than WES as it provides a more comprehensive testing without increasing costs significantly [41]. With regard to further genome wide tests such as chromosomal microarrays the performance of WGS for CNV detection has been shown to be at least comparable [39]. However, in the current literature no appropriate analytical validation of WGS for pharmacogenomic profiling has been addressed. Linderman et al. (2014) provided an evaluation of the analytical performance of WES and WGS in general using five reference samples and technical replicates, by assessing the reproducibility (intra-run, inter-run, inter-mode and inter-machine), the concordance with microarrays and Sanger Sequencing and the sensitivity. Here, for WES and WGS the focus was on sensitive and precise as possible variant calling over the target region and thereby the determination of bases for which appropriate requirements were not met. Here, the performance analysis was based on ranges recommended by The American College of Medical Genetics and Genomics [5].

However, short read based NGS currently applied for WES or WGS can be prone to coverage bias in GC rich regions also due to a dependence on PCR for template amplification and therefore may miss hidden genetic variation in numerous genomic regions with high GC content. Therefore, disruptions of relevant genes with such GC rich regions or impairments of dosage-sensitive genes leading to dosage change may remain undetected in patients assumed to have a genetic disorder. Many structural variants and indels undetected by short read based NGS were detected by long read sequencing in recent studies [42–44].

However, the low throughput in the application of platforms such as Nanopore ONT and PacBio prohibits cost-effective deep sequencing without enrichment of the target sequence of interest [45]. Nevertheless, barcoding and multiplexing of samples has been reported to reduce per sample expenses in *CYP2D6* SMRT sequencing and enable cost comparability to commercial genotyping assays. Moreover, full gene SMRT provides further *CYP2D6* variant information such as e.g. the characterization of CNVs, sub allele resolution and novel tandem arrangement which may be convenient for routine clinical testing [23].

Also in long read sequencing PCR based enrichment can introduce phasing errors [46] and amplification bias and also leads to a loss of information on native modifications. CRISPR-Cas-based enrichment strategies such as those for Nanopore Cas9-targeted sequencing (nCATS) can prevent such bias and information loss. Still, a relatively high amount of native DNA is needed and limitations in the detection of some SNVs remain. For the performance of this application, however, it is assumed that identification of SNVs will progress with accelerating improvements of algorithms for base- and variant-calling in future and will also increase performance for mutation surveillance [47].

Furthermore, many recent publications indicate that these evolving techniques hold considerable promise for clinical application [48–50]. However, for third generation sequencing with regard to drug metabolizing enzymes only one study providing validation results and performance comparisons with currently FDA cleared or approved nucleic acid based tests was detected via our literature screening based on the keyword search. The study detected was however based on low numbers of tested samples. Still, it provides a promising outlook for third generation sequencing technologies to detect variation also in the complex *CYP2D6* locus.

Currently, WES and WGS are performed based on second generation sequencing techniques as the emerging techniques in third generation sequencing are characterized by a high error rate. However, third generation sequencing provides a promising utility for the analysis of structurally complex genomic regions and may become a helpful tool for the assessment of the whole genome for clinical use [42, 51]. Thus, they are increasingly provided by commercial service laboratories, currently however for research evaluations only [52, 53].

A limitation of the present assessment is that other suitable keywords such as performance or concordance were not applied in this literature search. Therefore, several publications focusing on analytical validation in terms of analytical performance may have been missed. Another limitation is that NGS based diagnostic tests applied as clinical assays are mainly validated and applied in laboratories as LDT and documented data on analytical validation is not publicly available [6, 54]. In 2017, the FDA published a discussion paper on the oversight of LDTs. It addressed also an increase of transparency in terms of providing publicly available information on analytical and clinical validity of LDTs. However, a final guidance was not established [55]. Due to the new in vitro diagnostic regulation in the EU it will be mandatory for laboratories to provide appropriate performance evaluations to the regulatory authorities. The establishment of transparency in terms of publicly available information on the validity of laboratory developed and applied NGS technologies is not addressed by the new IVDR in the EU [3].

## Conclusions

Clinical NGS and knowledge in pharmacogenomics are evolving rapidly. However, current studies evaluating NGS-based test performance for pharmacogenetic profiling mainly provide concordance assessments with orthogonal methods without considering further relevant analytical performance characteristics recommended by the FDA or required according to the CAP and CLIA guidance and in the EU according to the IVDR. For the use of next generation sequencing applications such as WES, WGS and also third generation sequencing for pharmacogenetic profiling in the clinical setting, a more comprehensive analytical performance analysis including at least the recommended performance characteristics is needed. However, it became clear that for publication of such assessments a standard in reporting on analytical validation of NGS based tests is not in place and may be increasingly necessary to facilitate the implementation of NGS tests in clinical use. Feasibility evaluations to extract clinically important pharmacogenetic information from sequencing data obtained by diagnostic testing for other clinical conditions suggest a potential to reuse relevant information for therapy management. Therefore, regulatory guidance to establish analytical validation requirements also for such approaches may be necessary.

### Electronic supplementary material

Below is the link to the electronic supplementary material.

Supplementary Material 1

Supplementary Material 2

Supplementary Material 3

## Data Availability

Data supporting reported results are provided in Supplementary material 1 and 2.

## Abbreviations

CAP: College of American Pathologists
CE: Conformité Européenne
CLIA: Clinical Laboratory Improvement Amendments
CLSI: Clinical and Laboratory Standards Institute
CNV: Copy number variation
CYP: Cytochrome P450
EU: European Union
FDA: Food and Drug Administration
IVD: In Vitro Diagnostic
IVDR: In Vitro Diagnostic Medical Devices Regulation
LDT: Laboratory developed test
NGS: Next generation sequencing
NPA: Negative percent agreement
ONT: Oxford Nanopore Technologies
PacBio: Pacific Biosciences
PCR: Polymerase chain reaction
PGx: Pharmacogenomics
PPA: Positive percent agreement
PPV: Positive predictive value
SMRT: Single-molecule real-time
UGT1A1: UDP-Glucuronosyltransferase 1 Polypeptid A1
USA: United States of America
VKORC1: Vitamin K epOxide Reductase Complex Subunit 1
WES: Whole exome sequencing
WGS: Whole genome sequencing

## References

[1] Ritzhaupt A, Hayes I, Ehmann F (2020). Implementing the EU in vitro diagnostic regulation – a european regulatory perspective on companion diagnostics. Expert Rev Mol Diagn.

[2] Luh F, Yen Y (2018). FDA guidance for next generation sequencing-based testing: balancing regulation and innovation in precision medicine. NPJ Genom Med.

[3] COUNCIL EPA *REGULATION (EU) 2017/746 OF THE EUROPEAN PARLIAMENT AND OF THE COUNCIL of 5 April 2017 on in vitro diagnostic medical devices and repealing Directive 98/79/EC and Commission Decision 2010/227/EU* in *Official Journal of the European Union*

[4] Administration USFD (2021) *Overview of IVD Regulation*. [cited 2023 10.02.2023]; Available from: https://www.fda.gov/medical-devices/ivd-regulatory-assistance/overview-ivd-regulation#4

[5] Linderman MD (2014). Analytical validation of whole exome and whole genome sequencing for clinical applications. BMC Med Genom.

[6] Karlovich CA, Williams PM (2019). Clinical applications of next-generation sequencing in Precision Oncology. Cancer J.

[7] Jennings LJ (2017). Guidelines for validation of Next-Generation sequencing-based oncology panels: a Joint Consensus Recommendation of the Association for Molecular Pathology and College of American Pathologists. J Mol Diagn.

[8] Administration USFD (2018) *Considerations for Design, Development, and Analytical Validation of Next Generation Sequencing (NGS) - Based In Vitro Diagnostics (IVDs) Intended to Aid in the Diagnosis of Suspected Germline Diseases*. [cited 2022 22.08.2022]; Available from: https://www.fda.gov/regulatory-information/search-fda-guidance-documents/considerations-design-development-and-analytical-validation-next-generation-sequencing-ngs-based

[9] Administration USFD (2023) *List of Cleared or Approved Companion Diagnostic Devices (In Vitro and Imaging Tools)*. 02/14/2023 [cited 2023 23.02.2023]; Available from: https://www.fda.gov/medical-devices/in-vitro-diagnostics/list-cleared-or-approved-companion-diagnostic-devices-in-vitro-and-imaging-tools

[10] Lanillos J (2022). Clinical pharmacogenetic analysis in 5,001 individuals with diagnostic exome sequencing data. npj Genomic Medicine.

[11] Caspar SM (2021). Potential of whole-genome sequencing-based pharmacogenetic profiling. Pharmacogenomics.

[12] van der Lee M (2020). Repurposing of Diagnostic whole exome sequencing data of 1,583 individuals for clinical pharmacogenetics. Clin Pharmacol Ther.

[13] Schwarz UI, Gulilat M, Kim RB (2019) The role of Next-Generation sequencing in Pharmacogenetics and Pharmacogenomics, vol 9. Cold Spring Harb Perspect Med, 2 10.1101/cshperspect.a033027 PMC6360866 29844222

[14] Profaizer T (2020). Clinical utility of next generation sequencing based HLA typing for disease association and pharmacogenetic testing. Hum Immunol.

[15] Pulley JM (2012). Operational implementation of prospective genotyping for personalized medicine: the design of the Vanderbilt PREDICT project. Clin Pharmacol Ther.

[16] Rasmussen-Torvik LJ (2014). Design and anticipated outcomes of the eMERGE-PGx project: a multicenter pilot for preemptive pharmacogenomics in electronic health record systems. Clin Pharmacol Ther.

[17] van der Wouden CH (2017). Implementing pharmacogenomics in Europe: design and implementation strategy of the ubiquitous Pharmacogenomics Consortium. Clin Pharmacol Ther.

[18] Huebner T, Steffens M, Scholl C (2022). Molecular Genetic Techniques in Biomarker Analysis relevant for drugs centrally approved in Europe. Mol Diagn Ther.

[19] Cousin MA (2017). Pharmacogenomic findings from clinical whole exome sequencing of diagnostic odyssey patients. Mol Genet Genomic Med.

[20] Lanillos J (2022). Clinical pharmacogenetic analysis in 5,001 individuals with diagnostic exome sequencing data. NPJ Genom Med.

[21] Administration USFD, Nucleic Acid Based T (2023) [cited 2022 12.08.2022]; Available from: https://www.fda.gov/medical-devices/in-vitro-diagnostics/nucleic-acid-based-tests

[22] Carvalho Henriques B (2021). Methodology for clinical genotyping of CYP2D6 and CYP2C19. Translational Psychiatry.

[23] Qiao W (2016). Long-read single Molecule Real-Time full gene sequencing of cytochrome P450-2D6. Hum Mutat.

[24] Tang NY (2021). Validation of a large custom-designed Pharmacogenomics Panel on an array genotyping platform. J Appl Lab Med.

[25] Buermans HP (2017). Flexible and scalable full-length CYP2D6 long Amplicon PacBio sequencing. Hum Mutat.

[26] Chua EW (2016). Cross-Comparison of Exome Analysis, Next-Generation sequencing of amplicons, and the iPLEX(®) ADME PGx panel for pharmacogenomic profiling. Front Pharmacol.

[27] Hamilton A (2016). Concordance between whole-exome sequencing and clinical Sanger sequencing: implications for patient care. Mol Genet Genomic Med.

[28] Yang W (2016). Comparison of genome sequencing and clinical genotyping for pharmacogenes. Clin Pharmacol Ther.

[29] Beaubier N (2019). Clinical validation of the tempus xT next-generation targeted oncology sequencing assay. Oncotarget.

[30] Ng D (2017). Assessing the capability of massively parallel sequencing for opportunistic pharmacogenetic screening. Genet Med.

[31] Klein K (2019). A New Panel-Based next-generation sequencing method for ADME genes reveals Novel Associations of Common and Rare Variants with expression in a human liver cohort. Front Genet.

[32] Ramudo-Cela L (2020). Development and validation of a next-generation sequencing panel for clinical pharmacogenetics. Farmacia hospitalaria: organo oficial de expresion cientifica de la Sociedad Espanola de Farmacia Hospitalaria.

[33] Gulilat M (2019). Targeted next generation sequencing as a tool for precision medicine. BMC Med Genomics.

[34] Lee SB (2022). ClinPharmSeq: a targeted sequencing panel for clinical pharmacogenetics implementation. PLoS ONE.

[35] Silver A et al (2022) *Technical performance of a 430-Gene Preventative Genomics assay to identify multiple variant types Associated with Adult-Onset monogenic conditions, susceptibility loci, and pharmacogenetic insights*. J Pers Med, 12(5) 10.3390/jpm12050667 PMC9147210 35629091

[36] Xiang D et al (2019) *Comparing PyroMark Q24 pyrosequencing and MALDI-TOF MS for identification of CYP2D6*10*. Clin Lab, 65(5) 10.7754/Clin.Lab.2018.180909 31115220

[37] (CAP), C.o.A.P. Next Generation Sequencing (NGS) Worksheets (2018) [cited 2023; Available from: https://www.cap.org/member-resources/precision-medicine/next-generation-sequencing-ngs-worksheets

[38] Centers for Disease Control and Prevention (CDC) and Centers for Medicare & Medicaid Services (CMS), H., Medicare, Medicaid, and CLIA Programs; Laboratory Requirements Relating to Quality Systems and Certain Personnel Qualifications. A Rule by the Centers for Medicare & Medicaid Services and the Centers for Disease Control and Prevention on 01/24/ (2003) C.f.D.C.a.P. Centers for Medicare & Medicaid Services, Editor. 2003: Federal Register. p. 3639–3714 12545998

[39] Marshall CR (2020). Best practices for the analytical validation of clinical whole-genome sequencing intended for the diagnosis of germline disease. npj Genomic Medicine.

[40] Rehm HL (2013). ACMG clinical laboratory standards for next-generation sequencing. Genet Med.

[41] Caspar SM et al (2020) *Added value of clinical sequencing: WGS-Based profiling of Pharmacogenes*. Int J Mol Sci, 21(7) 10.3390/ijms21072308 PMC7178228 32225115

[42] Mantere T, Kersten S, Hoischen A (2019). Long-read sequencing emerging in Medical Genetics. Front Genet.

[43] Xu L (2023). Long-read sequencing identifies novel structural variations in colorectal cancer. PLoS Genet.

[44] Mahmoud M (2019). Structural variant calling: the long and the short of it. Genome Biol.

[45] Yang Y (2017). Sequencing the CYP2D6 gene: from variant allele discovery to clinical pharmacogenetic testing. Pharmacogenomics.

[46] Rubben K (2022). Cas9 targeted nanopore sequencing with enhanced variant calling improves CYP2D6-CYP2D7 hybrid allele genotyping. PLoS Genet.

[47] Gilpatrick T (2020). Targeted nanopore sequencing with Cas9-guided adapter ligation. Nat Biotechnol.

[48] Shahandeh A et al (2016) *Advantages of array-based Technologies for pre-emptive Pharmacogenomics Testing*. Microarrays (Basel), 5(2) 10.3390/microarrays5020012 PMC5003488 27600079

[49] Merker JD (2018). Long-read genome sequencing identifies causal structural variation in a mendelian disease. Genet Med.

[50] Logsdon GA, Vollger MR, Eichler EE (2020). Long-read human genome sequencing and its applications. Nat Rev Genet.

[51] Fujimoto A (2021). Whole-genome sequencing with long reads reveals complex structure and origin of structural variation in human genetic variations and somatic mutations in cancer. Genome Med.

[52] GmbH LB (2023) *Genomics*. [cited 2023 20.01.2023]; Available from: https://www.lifeandbrain.com/en/research-development/genomics/

[53] Technologies ON (2023) 20.01.2023] *Whole-genome sequencing with nanopore technology*. [cited ; Available from: https://nanoporetech.com/applications/whole-genome-sequencing

[54] Spitzenberger F et al (2022) Laboratory-Developed tests: design of a Regulatory Strategy in Compliance with the International State-of-the-art and the regulation (EU) 2017/746 (EU IVDR [In Vitro Diagnostic Medical device Regulation]), vol 56. Therapeutic Innovation & Regulatory Science, pp 47–64. 1 10.1007/s43441-021-00323-7 PMC8294224 34291407

[55] ADMINISTRATION USFD (2014) *Laboratory Developed Tests - Discussion Paper*. [cited 2023 10.01.2023]; Available from: https://www.fda.gov/media/102367/download

