# Supplementary material for: Current status of the analytical validation of next generation sequencing applications for pharmacogenetic profiling
Source: Mol Biol Rep. 2023 Oct 3;50(11):9587–99. doi: 10.1007/s11033-023-08748-z (PMC10635985; doi:10.1007/s11033-023-08748-z)
Supplement: Supplementary file 3 — Supplementary Material 3 [file 11033_2023_8748_MOESM3_ESM.docx]

**Current status of the analytical validation of Next Generation Sequencing applications for pharmacogenetic profiling**

Molecular Biology Reports

Tatjana Huebner^1^, Michael Steffens^1^, Catharina Scholl^1^

^1^Research Division, Federal Institute for Drugs and Medical Devices, Bonn, North Rhine-Westphalia, Germany

Correspondence to Dr. Tatjana Hübner

Tatjana.Huebner@bfarm-research.de

Supplementary material 3

Table 1 Recommendation of criteria for analytical validation of NGS (IVD)

|  | **FDA (NGS)[1]** | **CAP/ CLSI (NGS)[2]** | **CLIA (IVD in general)[3]** | **IVDR (IVD in general)[4]** |
| --- | --- | --- | --- | --- |
| **Precision** | | | | |
| Reproducibility | ✓ | ✓ | (✓) Precision (not specified) | ✓ |
| Repeatability | ✓ | ✓ | (✓) Precision (not specified) | ✓ |
| Accuracy | | | | |
| PPA (or analytical sensitivity) | ✓ | ✓ | (✓) accuracy (PPA not specified) | ✓ (analytical sensitivity) |
| NPA (or analytical specificity) | ✓ | ✓ | (✓) accuracy (NPA not specified) | ✓ (analytical specificity) |
| TPPV | ✓ | ✓ | (✓) accuracy (TPPV not specified) | (✓) accuracy (TPPV not specified) |
| **Specificity** | | | | |
| Interference | ✓ | ✓ | ✓ (interfering substances) | ✓ |
| Cross-Reactivity | ✓ | ✓ | ✓ (interfering substances) | ✓ |
| Cross-Contamination | ✓ |  |  |  |
| **Limit of detection (LOD)** | ✓ | ✓ | Analytical sensitivity (LOD not specified) | ✓ |
| **Further criteria** | To be established adequate to performance needs | - robustness /guard-banding - reportable range - reference interval | - reportable range - reference intervals (normal values). - any other performance characteristic required for test performance. | - trueness (bias) - measuring range - determination of appropriate criteria for specimen collection and handling - limit of quantitation - linearity - cut-off |

CAP: College of American Pathologists, CLSI: Clinical and Laboratory Standards Institute, FDA: Food and drug administration, IVD: In vitro diagnostic, NPA: Negative Percent Agreement, PPA: Positive Percent Agreement, TPPV: Technical Positive Predictive Value

**References**

1. Administration, U.S.F.D. *Considerations for Design, Development, and Analytical Validation of Next Generation Sequencing (NGS) - Based In Vitro Diagnostics (IVDs) Intended to Aid in the Diagnosis of Suspected Germline Diseases*. 2018 [cited 2022 22.08.2022]; Available from: <https://www.fda.gov/regulatory-information/search-fda-guidance-documents/considerations-design-development-and-analytical-validation-next-generation-sequencing-ngs-based>.

2. (CAP), C.o.A.P. *Next Generation Sequencing (NGS) Worksheets*. 2018 [cited 2023; Available from: <https://www.cap.org/member-resources/precision-medicine/next-generation-sequencing-ngs-worksheets>.

3. Centers for Disease Control and Prevention (CDC) and Centers for Medicare & Medicaid Services (CMS), H., *Medicare, Medicaid, and CLIA Programs; Laboratory Requirements Relating to Quality Systems and Certain Personnel Qualifications. A Rule by the Centers for Medicare & Medicaid Services and the Centers for Disease Control and Prevention on 01/24/2003*, C.f.D.C.a.P. Centers for Medicare & Medicaid Services, Editor. 2003: Federal Register. p. 3639-3714.

4. COUNCIL, E.P.A., *REGULATION (EU) 2017/746 OF THE EUROPEAN PARLIAMENT AND OF THE COUNCIL of 5 April 2017 on in vitro diagnostic medical devices and repealing Directive 98/79/EC and Commission Decision 2010/227/EU* in *Official Journal of the European Union*.
